# A cautionary tale about the use of colony-forming efficiency as a proxy for the survival of mesenchymal stem cells

**DOI:** 10.1186/s13287-020-01805-5

**Published:** 2020-07-16

**Authors:** Kim O’Connor

**Affiliations:** 1 Department of Chemical and Biomolecular Engineering, School of Science and Engineering, Tulane University, 6823 Saint Charles Ave., Boggs Center Room 300, New Orleans, LA 70118 USA; 2 Center for Stem Cell Research and Regenerative Medicine, School of Medicine, Tulane University, New Orleans, LA USA

**Keywords:** Colony-forming unit assay, Survival, Mesenchymal stem cells

## Abstract

Colony-forming efficiency is a time-honored metric of the proliferation potential of mesenchymal stem cells (MSCs). This commentary raises a concern about the practice of using colony-forming efficiency as a proxy for cell survival. A recently published study from my laboratory investigated this issue. A marker of cellular aging, CD264, was employed to separate human bone marrow MSCs into populations of CD264^−^ cells and culture-matched, aging CD264^+^ cells with high and low colony-forming efficiency, respectively. In vitro cell survival was evaluated with a single-cell assay; in vivo survival by bioluminescence imaging of MSCs attached to scaffolds that were implanted ectopically in immunodeficient mice. In our study, in vitro and in vivo survival of the MSC populations was independent of colony-forming efficiency. This finding indicates that caution should be exercised before using colony-forming efficiency as an indirect metric of cell survival. Direct measurement of survival may be required. Awareness of this issue should foster a robust experimental design and, thereby, facilitate the translation of MSC research into clinical practice.

## Background

The colony-forming unit (CFU) assay is a popular method to assess the proliferation potential of mesenchymal stem cells (MSCs). Friedenstein et al. were the first to discover MSCs by their characteristic ability to form discrete fibroblast colonies [1]. The CFU assay measures the efficiency by which MSCs form colony units when plated at clonogenic levels in monolayer culture on tissue culture plastic. Today, colony-forming efficiency is frequently employed for quality assessment of MSC preparations used in preclinical research and clinical trials [2, 3].

This commentary raises a concern about the practice of using colony-forming efficiency as an indirect measure of MSC survival. As discussed below, the underlying assumption of equivalence between these two parameters is invalid in some instances. A recently published study from my laboratory provides illustrative examples [4]. In such cases, measurements of colony-forming efficiency can lead to misinterpretation of experimental data and spurious conclusions about MSC survival. Instead, direct measurement of cell survival is warranted. Given the popularity of the CFU assay, its misuse has the potential to slow the translation of MSC research into clinical practice. The goal of highlighting this issue here is to promote rigorous MSC research and, in turn, accelerate the development and manufacturing of MSC therapies.

## In vitro and in vivo survival

**Fig. 1:**
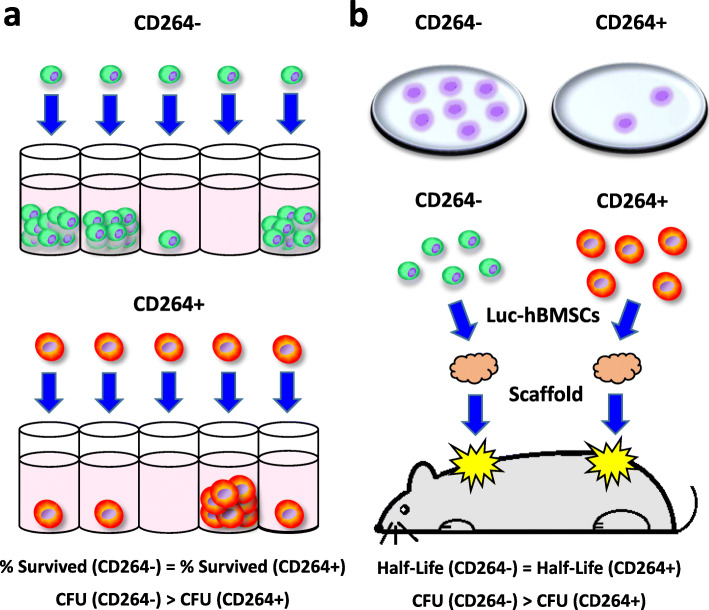
Illustration of the discrepancy between the colony-forming efficiency and survival of CD264^+/−^ populations of hBMSCs. The two populations were generated by fluorescence-activated cell sorting based on the expression of CD264, a marker of cellular aging. **a** In vitro survival and colony-forming efficiency was resolved at the single-cell level by inoculating multi-well plates via limiting dilution. In wells inoculated with single cells, culture-matched CD264^−^ and CD264^+^ populations had a comparable percentage of surviving cells after a week of culture, as measured by cell attachment; however, fewer of the aging CD264^+^ cells formed colonies > 10 cells during the same period. **b** In vivo survival of the two populations was evaluated by bioluminescence imaging for a month after the cells were implanted subcutaneously on the dorsum of immunodeficient mice. Prior to implantation, hBMSCs were transduced with a luciferase, sorted into CD264^−^ and CD264^+^ populations, and attached to ceramic scaffolds. For each sorted population, colony-forming efficiency was measured 2 weeks after the cells were plated at clonogenic levels into 10-cm culture dishes. The luminescence half-life was similar for culture-matched CD264^−^ and CD264^+^ populations despite a lower efficiency for CD264^+^ cells to form colonies > 50 cells. CFU, colony-forming unit; hBMSCs, human bone marrow mesenchymal stem cells; Luc, luciferase

Many stem cell scientists recognize that cell survival is only one of several factors that contribute to colony formation [5]. Some researchers, however, employ colony formation as an indirect measure of in vitro cell survival [6, 7]. This practice of using colony formation as a proxy for survival yields a false-negative result when a cell survives in culture but is unable to proliferate into a colony. In this scenario, colony-forming efficiency underestimates the percentage of surviving cells. Recently, my research group published an illustrative example with human bone marrow MSCs (hBMSCs) [4]. Each culture was separated into two populations by fluorescence-activated cell sorting based on surface expression of CD264. We previously identified CD264 as a marker of cellular aging whose expression is upregulated in hBMSCs during serial passage and is correlated to elevated levels of senescence-associated β-galactosidase [8]. Aging CD264^+^ hBMSCs formed colonies less efficiency relative to their CD264^−^ counterpart, but the two populations had comparable in vitro survival at the single-cell level (Fig. 1a) [4]. Reliance on only colony formation as a metric of survival would have produced false-negative results in this case. The use of a single-cell assay, like the one in our study, is an effective method to evaluate both in vitro survival and colony formation.

Others have suggested that in vitro colony-forming efficiency may be a predictive metric of in vivo MSC survival [9]. Our findings do not support this supposition. In the study described above, we compared the survival of CD264^+/−^ hBMSCs attached to ceramic scaffolds, which were implanted subcutaneously in immunodeficient mice [4]. Bioluminescence imaging revealed that matched implants of CD264^−^ and CD264^+^ hBMSCs from the same culture had a similar in vivo half-life despite a lower colony-forming efficiency for the aging CD264^+^ cells (Fig. 1b) [4]. These findings cast doubt on the utility of colony-forming efficiency as a metric of in vivo survival. Other in vitro assays are needed that have greater predictive value and mimic the stresses that MSC encounter upon implantation [10].

## Conclusions

The CFU assay is an important tool for quality assessment of MSCs in research and biomanufacturing, but there are limits to its use. Caution is warranted when considering colony-forming efficiency as a proxy for the in vitro and in vivo survival of MSCs. Examples were provided where this practice is invalid. Awareness of this problem should promote more robust survival research and expedite its translation into effective MSC therapies.

## Data Availability

Not applicable

## Abbreviations

CFU: Colony-forming unit
MSCs: Mesenchymal stem cells
hBMSCs: Human bone marrow mesenchymal stem cells
